# Naturally Occurring Polymorphisms of the Mouse Gammaretrovirus Receptors CAT-1 and XPR1 Alter Virus Tropism and Pathogenicity

**DOI:** 10.1155/2011/975801

**Published:** 2011-10-23

**Authors:** Christine A. Kozak

**Affiliations:** Laboratory of Molecular Microbiology, National Institute of Allergy and Infectious Diseases, Bethesda, MD 20892-0460, USA

## Abstract

Gammaretroviruses of several different host range subgroups have been isolated from laboratory mice. The ecotropic viruses infect mouse cells and rely on the host CAT-1 receptor. The xenotropic/polytropic viruses, and the related human-derived XMRV, can infect cells of other mammalian species and use the XPR1 receptor for entry. The coevolution of these viruses and their receptors in infected mouse populations provides a good example of how genetic conflicts can drive diversifying selection. Genetic and epigenetic variations in the virus envelope glycoproteins can result in altered host range and pathogenicity, and changes in the virus binding sites of the receptors are responsible for host restrictions that reduce virus entry or block it altogether. These battleground regions are marked by mutational changes that have produced 2 functionally distinct variants of the CAT-1 receptor and 5 variants of the XPR1 receptor in mice, as well as a diverse set of infectious viruses, and several endogenous retroviruses coopted by the host to interfere with entry.

## 1. Introduction

The various inbred strains of laboratory mice and wild mouse species differ in their susceptibility to mouse gammaretrovirus infection and to virus-induced diseases. Host resistance is due to numerous constitutively expressed antiviral factors that target specific stages of the retroviral life cycle. These host restriction factors can block entry, postentry uncoating and reverse transcription, trafficking, integration, assembly, and release [1]. The first step in the replicative cycle is entry, and this process relies on host-encoded receptors. Host cell factors that can interfere with virus entry include genetic variations of the cell receptor as well as other host factors such as envelope (Env) glycoproteins produced by endogenous retroviruses (ERVs).

Infectious mouse leukemia viruses (MLVs) of three subgroups have been isolated from laboratory mice, and these subgroups were initially defined by their species tropisms. The ecotropic MLVs (E-MLVs) infect only mouse or rat cells and use the amino acid transporter CAT-1 as receptor. The xenotropic MLVs (X-MLVs) infect cells of non-rodent species [2], and polytropic MLVs (P-MLVs) infect both mouse and non-rodent cells [3, 4]. The X-MLVs and P-MLVs together constitute the XP-MLVs and both use the XPR1 receptor [5–8].

 Receptor choice is determined by the N-terminal portion of the MLV Env, the receptor-binding domain (RBD) [9–11]. The E-MLVs and XP-MLVs both have Env subtypes that differ in their ability to use polymorphic variants of their cognate receptors, and some of these host-range variants, the “xenotropic” MLVs, are completely restricted in mouse cells [12]. Both receptors for the laboratory mouse MLVs, CAT-1 and XPR1, have naturally occurring variants responsible for specific virus resistance phenotypes. There are 2 functionally distinct variants of the CAT-1 receptor for E-MLVs [13], and there are 5 known variants of the XPR1 receptor for the XP-MLVs in mice [5, 14–17]. These variants are not only important host factors that can restrict infection, but also they can alter virus-receptor interactions in ways that influence virus-induced pathology. This paper will describe the functional variants of these 2 MLV receptors and describe their coevolution with MLV in virus-infected mouse populations. 

## 2. The CAT-1 Receptor for E-MLVs

**Figure 1:**
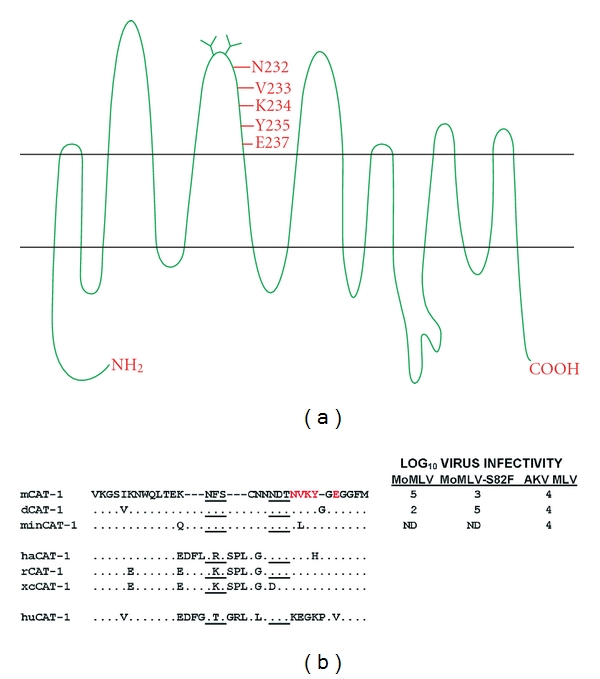
Predicted topology and sequence variation of the CAT-1 receptor for mouse ecotropic gammaretroviruses. (a) Putative topology identifies 14 predicted transmembrane domains. The third extracellular loop contains critical residues for receptor function (in red) and two N-linked glycosylation sites. (b) Sequence variation in the CAT-1 third extracellular loop. At the top are three sequence variants found in *Mus* with residues critical for entry in red. Virus infectivity of cells expressing these receptors is measured as the log_10_ titer of FFU/100 *μ*L of viral Env pseudotypes carrying the LacZ reporter gene; ND: not done. Consensus sites for N-glycosylation are underlined. CAT-1 sequence variation is shown for mouse CAT-1 variants mCAT-1 (NIH 3T3), dCAT-1 (*M. dunni*), and minCAT-1 (*M. minutoides*). E-MLV-infected *Mus* species *M. castaneus*, *M. molossinus*, *M. spicilegus*, and *M. musculus* are identical to mCAT-1 in the indicated region. Also shown are CAT-1 sequences for virus-susceptible species hamster (ha), rat (r), and XC rat cells (xc) and for virus-resistant human (hu).

The first gammaretrovirus receptor gene to be cloned was the CAT-1 receptor for E-MLVs [18]. This gene (gene symbol *Slc7a1*) encodes a glycoprotein with 14 putative transmembrane domains, and it functions as a cationic amino acid transporter [19, 20] (Figure 1(a)). Ten additional gammaretrovirus receptors have now been cloned; all of these gammaretrovirus receptors are multi-transmembrane proteins, and the receptors with known functions are all transporters of small solutes (reviewed in [21–26]). The human orthologue of mouse CAT-1 does not function as an E-MLV receptor, and the key sites in the mouse protein critical for virus entry lie in the third extracellular loop along with two consensus recognition sites for N-linked glycosylation [27, 28] (Figures 1(a) and 1(b)). CAT-1 is modified posttranslationally by glycosylation, and N-glycans are added to both of the CAT-1 loop 3 glycosylation sites [29]. All E-MLVs rely on the CAT-1 receptor for entry, although initial binding and the efficiency of entry may be influenced by other factors at the cell surface, such as heparin [30, 31].

**Figure 2:**
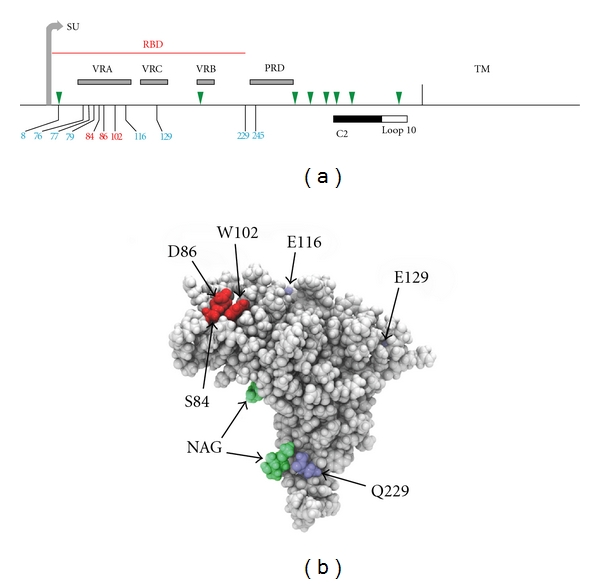
Structure of the FrMLV E-MLV Env gene. (a) Stick figure representation identifies the surface (SU) and transmembrane (TM) domains, the receptor binding domain (RBD) containing three variable regions (VRA, VRB, and VRC), and the proline-rich domain (PRD). Green triangles mark the N-linked glycosylation sites in SU. Vertical lines identify residues with roles in entry; the three residues in red form the binding pocket. The C-terminal segments designated C2 and loop10 have also been implicated in entry [42, 43]. (b) Surface representation of the FrMLV RBD (PDB ID 1AOL) [36], showing the location of the binding pocket (red), additional residues involved in entry (blue), and two N-linked glycosylation sites (green).

The E-MLV Env glycoprotein consists of surface (SU) and transmembrane (TM) subunits that are proteolytically cleaved from the same precursor protein and are linked by disulfide bonds. The SU protein has a 236 residue RBD at its N-terminal end that has 3 variable regions, and this is followed by a proline-rich hinge region and a C-terminal domain (Figure 2(a)). Entry is initiated by virus-receptor binding which precipitates a conformational change in Env that allows for subsequent fusion of viral and cellular membranes, a process that may involve cellular proteases [32]. The CAT-1 receptor recognition site is within the first variable domain (VRA) of the RBD. Three residues within the RBD VRA of the Friend E-MLV, FrMLV, have been identified as critical for entry (S84, D86, and W102) [33–35]. The crystal structure and functional analysis of the FrMLV RBD showed that these residues form a binding pocket on the structure's surface [35, 36] (Figure 2(b)). Several additional Env residues outside the binding pocket affect postbinding entry. Thus, the H8 residue in a conserved SPHQV motif near the SU N-terminus is necessary for fusion [37, 38] although residues at the other end of the MoMLV RBD, at positions 227 and 243 (equivalent to FrMLV sites 229 and 245), can substitute for H8 [39]. The proline-rich region is also involved in mediating postbinding conformational changes and fusion [40], and residues in two segments of the C-terminus of Env also have roles in fusion [41–43] (Figure 2(a)). 

## 3. Variants of the CAT-1 Receptor and E-MLV Env Affect Virus Entry

There has been no systematic attempt to screen for CAT-1 receptor variation in mice, but 3 sequence variants have been identified in *Mus* (Figure 1(b)). The prototype receptor, mCAT-1, was cloned from NIH 3T3 cells [18]. Two sequence variants have been identified in the wild mouse species *M. dunni* and *M. minutoides* [13, 44]. Limited testing suggests that the *M. minutoides* CAT-1 functions like the laboratory mouse mCAT-1 receptor, but the receptor of *M. dunni*, dCAT-1, differs from mCAT-1. *M. dunni* cells are relatively resistant to infection by Moloney E-MLV (MoMLV), although these cells are fully susceptible to other E-MLV isolates [13]. dCAT-1 differs from mCAT-1 by 4 residues, two of which are in the receptor determining third extracellular loop; one, I214V, is a substitution, and the second is a glycine insertion within the NVKYGE virus binding site [13] (Figure 1(b)). 

Two mutational changes in the MoMLV RBD VRA independently produce viruses that efficiently infect *M. dunni* cells: a replacement mutation, S82F, and introduction of two serine residues that are present in other E-MLV VRAs but absent in MoMLV (S76, S77 in FrMLV) (Figure 2(a)) [45]. The MoMLV S82F mutation site corresponds to S84 in Friend MLV, one of the 3 residues critical for virus binding and entry. The importance of this residue for virus tropism is underscored by the fact that MoMLV-S82F is poorly infectious in cells that carry mCAT-1 [45] (Figure 1(b)). 

E-MLVs can infect rodent species in addition to *Mus*, and CAT-1 receptor variants have been described in hamsters and rats (Figure 1(b)). Hamster cells are generally resistant to infection by E-MLVs, but some variants of FrMLV can infect these cells [46]. Infectivity of one such variant, PVC-211, was attributed to Env substitutions E116G and E129K [47] (Figure 2). Another FrMLV variant, F-S MLV, also inefficiently but reproducibly infects hamster cells; it was suggested that this tropism was influenced by two substitutions: S84A and S79N [48]. Restriction of some E-MLVs can result from complementary changes in the interacting sites of virus Env and the CAT-1 receptor; however, the MoMLV restriction associated with dCAT-1 is reproduced in human cells expressing this receptor [13], but not in ferret cells [49], suggesting that other cellular factors may also influence receptor function.

## 4. CAT-1 and Env Polymorphisms Associated with Pathogenicity

Polymorphisms that alter virus-receptor interactions can affect pathogenesis as well as entry. Cytopathic variants are common among the retroviruses that induce disease in their hosts, including HIV-1 as well as avian leukosis viruses and some pathogenic bovine and feline leukemia viruses [50–52]. These viruses can produce large multinucleated syncytia in cultures of susceptible cells. In contrast, mouse gammaretroviruses rarely produce syncytia although there are three exceptional cytopathic E-MLVs. The MoMLV variant, Spl574 and a FrMLV variant, F-S MLV, both induce syncytia and cell death in *M. dunni *cells [45, 48]. The third cytopathic virus, TR1.3, is a neuropathic FrMLV variant that also induces syncytia in SC-1 cells [33]. 

The cytopathicity of these 3 viruses is due to single amino acid substitutions at two of the 3 amino acids that form the receptor binding site. The cytopathicity of TR1.3 is due to W102G [33], and the cytopathicity of the other variants is due to different amino acid substitutions at the same critical Env residue: S82F in Spl574, and S84A in F-S MLV [45, 48]. Syncytium formation by Spl574 and F-S MLV is accompanied by the accumulation of large amounts of unintegrated viral DNA [48], a phenomenon which is also a hallmark of other cytopathic retroviruses and has been attributed to the absence of superinfection interference [53]. TR1.3 shows significantly reduced receptor binding avidity that correlates with its inability to block superinfection [54]. That cytopathicity is a consequence of altered receptor virus interactions is also supported by the fact, noted above, that MoMLV-S82F shows altered host range (Figure 1(b)) and also by the fact that syncytia formation by Spl574 is observed in cells of heterologous species expressing dCAT-1, but not mCAT-1 [49]. Thus, the cytopathicity of these 3 viruses in cultured cells and the neurovirulence of TR1.3 are governed by sequence differences in the viral *env* and, for 2 of these viruses, by corresponding differences in the CAT-1 receptor. 

Other polymorphisms of the E-MLV *env* can alter cell tropism and influence disease type. The thymotropism of radiation leukemia virus has been mapped to *env* [55], and several E-MLVs have neuropathogenic properties due to *env* polymorphisms. For example, TRM, a mutant variant of the neuropathic TR1.3, induces a different disease pathology resulting from reversion of the TR1.3 G102W mutation and a new Env mutation, S159P [56]. The most extensively studied neuropathic E-MLV is CasBrE, an isolate from California wild mice. Early studies mapped neurovirulence determinants to the CasBrE Env [57], and recent data indicate that CasBrE neuropathology is mediated by Env at two levels. First, the CasBrE Env targets the virus to cells within the CNS that express significant levels of CAT-1, and second, disease-associated spongiosis is induced by MLV-receptor-independent toxicity of this Env, determinants of which have not been identified but are presumably shared with other neurovirulent MLVs [58]. Env residues have also been implicated in the targeting of the neuropathic Friend PVC-211 variant to brain capillary endothelial cells [31]. This tropism is due to 2 mutations, E116G in VRA and E129K in VRC, mutations that also alter host range and interference properties [47, 59]. These findings indicate that specific replacement substitutions at different positions in the Env of pathogenic E-MLVs can affect receptor interactions, cell tropism, disease induction, and disease type.

## 5. The Role of Glycosylation in E-MLV Entry and Tropism

The retroviral Env is glycosylated, as are cellular proteins involved in entry. Many viruses use the glycans on cell surface glycoproteins as attachment factors [60], but glycosylation of the CAT-1 receptor is not required for virus entry. CAT-1 continues to support virus entry after both loop 3 N-glycan sites have been removed by mutagenesis [61]. However, host cell glycans can modulate entry of some E-MLVs. Thus, resistance of *M. dunni* cells to MoMLV, resistance of NIH 3T3 cells to Spl574, and resistance of primary rat fibroblasts and hamster cells to E-MLVs are relieved by inhibitors of glycosylation [49, 62–66]. It is not clear whether the responsible glycoprotein is CAT-1 or other host glycoproteins, like the secreted factor associated with resistance to gibbon ape leukemia virus in hamster cells [64]. There is, however, some evidence that the restriction of E-MLV infection in rat cells may be regulated by the glycosylation of rat CAT-1. The CAT-1 of rat XC sarcoma cells lacks one of the glycosylation sites found in the CAT-1 gene of other rat cells (Figure 1(b)), and heterologous cells expressing xcCAT-1 were found to be more susceptible to MoMLV than cells expressing rCAT-1 [67]. 

Glycosylation of the viral Env has been associated with altered infectivity of multiple viruses including retroviruses such as HIV-1 [68]. MLV Envs can have up to 9 N-linked glycans (Figure 2(a)), and while glycans are critical for the maturation and transport of Env [69], functional roles for the individual Env glycans are poorly defined. It has been shown that loss of MoMLV gs2 results in a virus that is temperature sensitive in Rat2 cells, loss of gs4 produces noninfectious virus lacking SU protein, and loss of gs7 alters fusion and infectivity [70–73]. Removal of either of the 2 glycosylation sites in the Env RBD, gs1 and gs2, can produce viruses restricted by *M. dunni* cells due to altered virus binding to dCAT-1, although E-MLVs differ in their reliance on these glycans [74, 75]. Thus, N-linked glycans on the viral Env are required for proper folding and can influence the entry process, while glycans are not needed for CAT-1 receptor processing or receptor function and have, at best, limited ability to modulate virus entry. 

## 6. CAT-1 and E-MLV Env Variation in Wild Mouse Species

**Figure 3:**
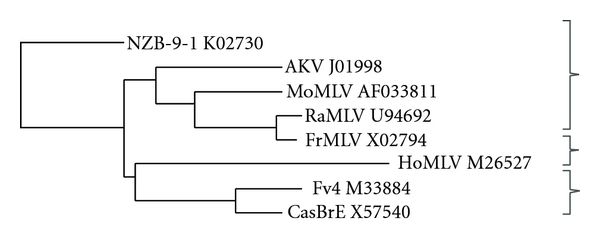
Phylogenetic tree of the Env genes of E-MLV gammaretroviruses. The tree includes laboratory mouse isolates FrMLV, MoMLV, and Rauscher MLV (RaMLV), the naturally occurring viruses AKV MLV, CasBrE, and HoMLV, and the Env gene of the *Fv4* restriction gene. The three related groups are bracketed. Sequences from GenBank were aligned using ClustalW2 and used to generate neighbour-joining trees. The X-MLV NZB-9-1 was included to root the tree.

Exposure to E-MLV gammaretroviruses occurred only recently in the evolution of *Mus* [76]. Although E-MLV ERVs are found in few of the 40 *Mus* species, wild mice carry three distinctive Env subtypes of E-MLVs (Figure 3). Sequence identity in SU*env* among these virus types is 70–77%. The first E-MLV type, the AKV E-MLVs of the laboratory mouse, is found as ERVs in multiple inbred strains [77]. Many of these proviruses are capable of producing infectious virus [78], and the widely used laboratory virus strains MoMLV, FrMLV, and Rauscher MLV are derived from AKV MLV [79] (Figure 3). Among the wild mouse species, AKV MLV ERVs are found in the Asian species *M. molossinus* and in *M. musculus* of Korea and China but not eastern Europe [76, 80]. A second E-MLV subtype was initially identified in California wild mice [81, 82]. Proviruses with this CasBrE Env type have also been found in the Asian species *M. castaneus,* and these virus-infected mice were likely introduced to California by passive transport from Asia [76, 80, 83, 84]. A third E-MLV subtype, HoMLV, was isolated from the eastern European species *M. spicilegus*, but is transmitted only as an exogenous virus [85]. 

The evidence indicates that these 3 E-MLV Env variants did not coevolve with receptor polymorphisms. Sequence comparisons indicate that the 3rd extracellular loop of the CAT-1 gene is invariant in wild-derived mice carrying these 3 E-MLVs: *M. castaneus*, *M. molossinus*, *M. spicilegus,* and *M. musculus* (GenBank Accession nos. JN226407–JN226410). The only known functional variant of this receptor in mice is dCAT-1 of *M. dunni*, and it is not clear if this variant arose in mice exposed to virus; the single available *M. dunni* sample is a cell line that does not carry E-MLV ERVs indicative of past infections [86], and it has not been determined if dCAT-1 is present in natural populations of *M. dunni* (now termed *M. terricolor*) or whether it originated by mutation in this cultured cell line. Sequence conservation of the receptor determining region of CAT-1 in *Mus* is not due to functional constraints as the third extracellular loops of the CAT-1 (SLC7A1) genes of various non-*Mus* species are quite variable (Figure 1(b)). Although E-MLVs can use CAT-1 receptor variants in non-*Mus* rodents, the replacement mutations in other mammalian species are incompatible with receptor function, thus limiting E-MLVs to rodents, a type of host range restriction that is not shared by other gammaretroviruses. The absence of polymorphism in the *Mus* CAT-1 gene, even in virus-infected wild mouse populations, is consistent with the conclusion that exposure to E-MLVs is very recent in *Mus *[76] and suggests that these mice rely on alternative survival strategies to limit the deleterious effects of infection, including posttranslational modification of receptor function, receptor interference, or postentry blocks in the retroviral lifecycle.

## 7. The XPR1 Receptor for XP-MLVs

**Figure 4:**
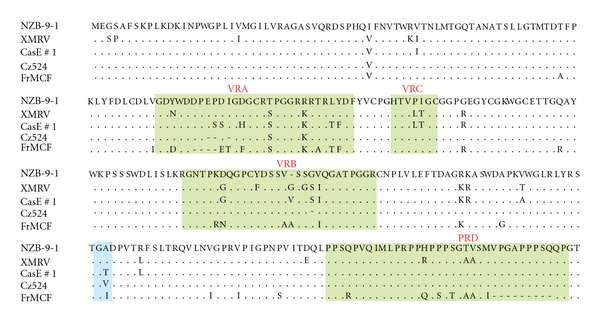
Alignment of the predicted amino acid sequences of the N-terminal portion of the Env sequences of various XP-MLVs. Included are the prototype NZB-9-1 X-MLV, the Friend FrMCF P-MLV, the wild mouse isolates CasE#1 and Cz524, and XMRV. Green blocks identify the three variable domains of the RBD and the PRD, and a blue block identifies two residues that influence species tropism [93].

Two subgroups of nonecotropic MLVs have been isolated from laboratory mice. These viruses were originally described as having distinct host ranges, but they use the same receptor, XPR1. X-MLVs and P-MLVs are both capable of infecting cells of nonrodent species, and although P-MLVs can efficiently infect mouse cells, X-MLVs were initially identified as incapable of infecting their natural hosts [2, 87, 88]. X-MLVs and P-MLVs are closely related viruses, and sequence differences in *env* and LTR are responsible for their differences in species tropism, for their nonreciprocal interference patterns, and for the pathogenicity of P-MLVs in mice [11, 89–92]. Although it is clear that the RBD VRA region is the major determinant of P-MLV and X-MLV host range [11], the critical VRA residues involved in XPR1 receptor recognition have not been identified, although 2 residues outside VRA can influence the ability of these viruses to infect cells of other mammalian species (Figure 4) [93]. Viruses in the XP-MLV family are highly variable in the Env segment containing the RBD (Figure 4), and the wild mouse viruses, CasE#1 and Cz524, show atypical host range patterns that distinguish them from prototypical P-MLVs and X-MLVs (Table 1) [16, 89, 94].

**Figure 5:**
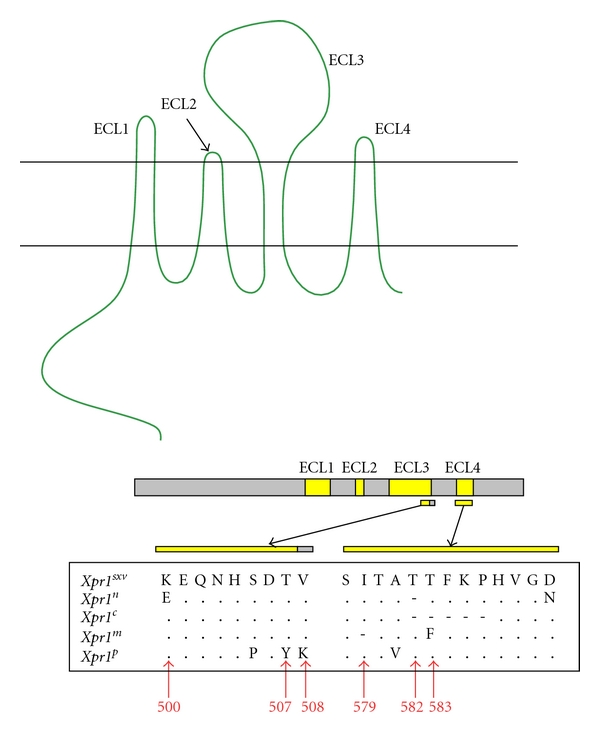
Predicted topology and sequence variation of the XPR1 receptor for XP-MLVs. At the top is shown the predicted structure with eight putative transmembrane domains and 4 extracellular loops (ECLs). The center diagram shows the relative locations of the 4 ECLs in the XPR1 protein, and the bottom shows sequence variation in the two ECLs involved in virus entry. Sequence is provided for the 5 functional XPR1 variants in *Mus*, and the red arrows indicate the 6 residues involved in entry.

The XPR1 receptor was originally described in laboratory mice as a P-MLV susceptibility gene [14]. Subsequent studies demonstrated that wild-mouse-derived cell lines, SC-1 and *M. dunni*, are susceptible to X-MLVs as well as P-MLVs [86, 96], while cells of the Asian species *M. castaneus* are resistant to P-MLVs [15]. That a single gene controls susceptibility to these two viruses was supported by the equivalent chromosome map locations of the genes controlling wild mouse susceptibility to X-MLVs and P-MLVs and by their cross-interference [5, 15, 89, 97]. The human and mouse *Xpr1* genes were cloned [6–8] and shown to encode a protein with 8 putative transmembrane domains (Figure 5). While a cellular function has not been assigned to XPR1, XPR1 is upregulated following activation of the NF-*κ*B RANKL-RANK signaling pathway [98] and its closest homologues in yeast (SYG1) and plants (PHO1) function in signal transduction and phosphate sensing and transport, respectively [8].

## 8. Naturally Occurring Variants of the XPR1 Receptor in *Mus*

**Figure 6:**
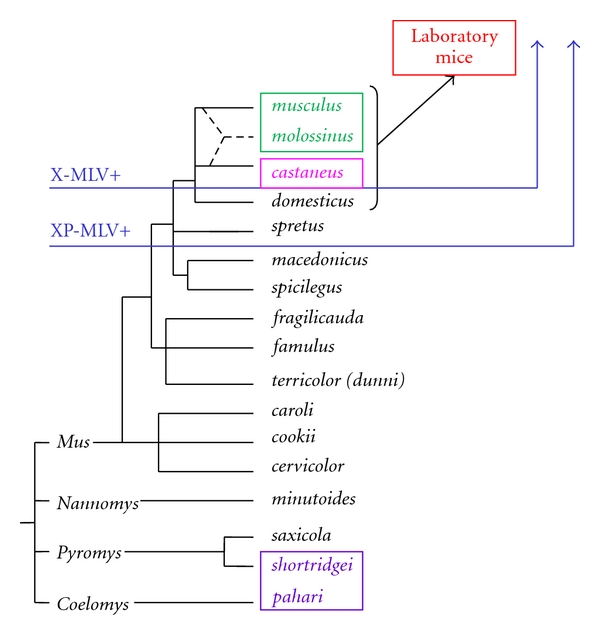
Phylogenetic tree of *Mus*. Blue arrows indicate the species that have acquired XP-MLV ERVs, and the subset that have predominantly X-MLVs. 4 colored boxes identify the mice carrying the 4 restrictive *Xpr1* alleles; all other species carry the permissive *Xpr1^sxv^*.

**Figure 7:**
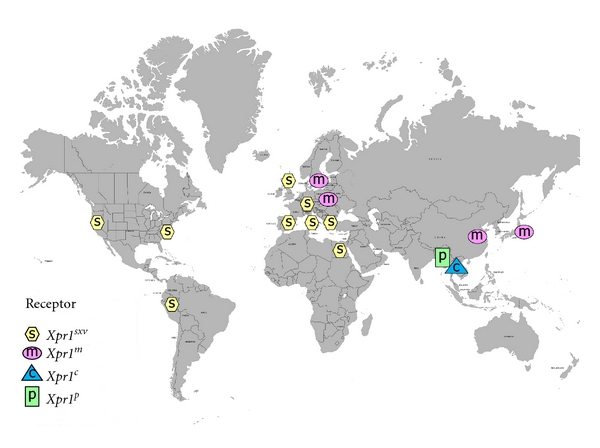
Geographic distribution of *Xpr1 *alleles in wild mouse populations.

The genus *Mus* includes about 40 species, and all available species have been screened for sequence and functional variants of *Xpr1 *(Figures 5 and 6). Of the 5 sequence variants found in wild mice, 4 show unique receptor phenotypes based on their ability to support entry of different virus isolates that rely on this receptor (Table 1). The most common receptor variant among wild mouse species was originally termed *Sxv *(susceptibility to xenotropic virus) [5]. This variant is found in many Asian species as well as western European house mice [17, 99], and mice with *Sxv* were introduced into the Americas by European immigrants and explorers (Figure 7). *Sxv* is also carried by several of the common inbred strains of laboratory mice [95]. *Sxv* is the most permissive of the *Xpr1* alleles and supports entry of all XP-MLV host range variants (Table 1). The second most geographically widespread *Xpr1* allele, *Xpr1^m^*, is found in two house mouse species, *M. musculus*, which ranges from central Europe to the Pacific, and *M. molossinus*, found in Japan [17]. This variant is highly restrictive, allowing inefficient entry of X-MLVs, while restricting all other XP-MLVs. A third allele, *Xpr1^c^*, is found in the southeast Asian mouse, *M. castaneus*, and is responsible for resistance to infection by P-MLVs [15, 99]. A fourth wild mouse *Xpr1* allele is restricted to the Asian species *M. pahari*; these mice are susceptible to X-MLVs and to CasE#1 [16] (Table 1).

There is a fifth mouse *Xpr1* variant, *Xpr1^n^*. The first of the receptor alleles to be identified, *Xpr1^n^,* was cloned from NIH 3T3 laboratory mouse cells [6–8]. This variant is responsible for the restrictive phenotype originally used to define the “xenotropic” host range subgroup, that is, viruses unable to infect cells of their home species [12]. *Xpr1^n^* is unusable by X-MLVs or by the 2 wild mouse virus isolates, although it supports entry by P-MLVs (Table 1). The origin of this laboratory mouse variant is unclear. Although *Xpr1^n^* is carried by the majority of common inbred mouse strains, it has not been found in any wild-trapped mouse [17, 95] (Figure 6). The common strains of laboratory mice were derived from colonies of fancy mice maintained by hobbyists, and they represent a mosaic of wild mouse species [100, 101]. Genomic analysis of multiple strains indicates that the predominant contributor to the laboratory mouse is *M. domesticus*, the western European mouse, with smaller contributions from *M. musculus* and *M. castaneus *[101]. Although this would suggest an *M. domesticus* origin for the laboratory mouse receptor allele, *M. domesticus* mice trapped in disparate locations in Europe and the Americans all carry the permissive allele [17] suggesting that *Xpr1^n^* arose and/or was selected in the fancy mice (Figure 7).

## 9. Genetic Basis of XPR1 Functional Polymorphism

Initial studies on *Xpr1* receptor function focused on sequence differences between the phenotypic variants identified in NIH 3T3 cells (*Xpr1^n^*) and *M. dunni* (*Xpr1^sxv^*) [99]. Two critical amino acids were identified for X-MLV entry that lie in different putative extracellular loops (Figure 5). The restrictive *Xpr1^n^* carries a substitution, K500E, in its third extracellular loop (ECL3), and a deletion, T582Δ, in the fourth loop (ECL4). Corrective mutations at either of these sites produce functional receptors for X-MLVs without compromising P-MLV receptor function [99]. Subsequent studies on the mouse receptor showed that these 2 critical residues are not equivalently used by the XP-MLVs, as CasE#1 can use *Xpr1^n^*-Δ582T but not *Xpr1^n^*-E500K [16]. Mutational analysis of other polymorphic sites in the various *Mus Xpr1s* identified residues at additional sites that modulate virus entry: ECL3 positions 500, 507, and 508 and ECL4 positions 579 and 583 (Figure 5) [17, 94].

**Figure 8:**
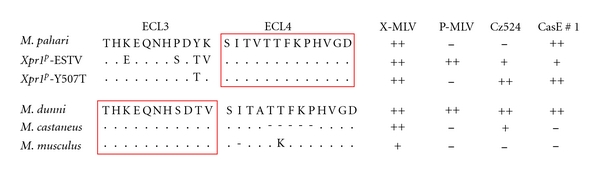
The entry of various XP-MLVs is affected by sequence variation in ECL3 or ECL4 suggesting that these domains form a single receptor site.

Although it is possible that the XPR1 protein carries two separate receptor determinants on ECL3 and ECL4 [102], it is more likely that the key residues in ECL3 and ECL4 form a single virus attachment site. The various viruses that use XPR1 for entry are sensitive to mutational changes in both ECL3 and ECL4 [16, 17, 94] (Figure 8). Thus, P-MLVs and the wild mouse viruses CasE#1 and Cz524 show different patterns of infectivity for* Xpr1^p^* mutants that have substitutions in ECL3 but identical ECL4 sequences. These same viruses also differ in their infectivity for cells with *Xpr1^m^*, *Xpr1^c^,* and *Xpr1^sxv^*, which have identical ECL3 sequences but different deletions in ECL4. The involvement of residues in multiple receptor domains is also characteristic of other retrovirus receptors [103]. While the different domains required for these other retroviral receptors can have distinctive roles in virus attachment and entry [104, 105], this division of labor has not yet been shown to be the case for the XPR1 ECL3 and ECL4 domains.

## 10. P-MLV Entry That Is Independent of the XPR1 Receptor

Although it is clear that MLV entry is typically mediated by specific cell surface receptors, some MLVs are capable of bypassing the need for their cognate receptors and can infect cells that lack receptors and may also be able to infect cells in which those receptors are downregulated by superinfection [106, 107]. Such alternative entry mechanisms seem to be particularly important for P-MLVs, viruses that are less able to establish effective superinfection immunity against further infection [99, 108] either because they may have lower binding affinity for the XPR1 receptor than the X-MLVs or because Env-bound receptors may recycle rapidly into acidic compartments where the Env-receptor complex is disrupted allowing the freed receptor to recycle back to the plasma membrane. This ineffective or delayed establishment of interference to exogenous infection has been linked to the massive accumulation of viral DNA in P-MLV-infected mice [109] and to the ability of P-MLVs to induce cytopathic responses in mink lung cells in which superinfection induces an ER stress response and apoptosis [4, 110, 111].

Infectious P-MLVs arise in preleukemic tissues of mice with high levels of E-MLVs. P-MLVs have recombinant genomes in which *env* sequences are derived from endogenous polytropic or modified polytropic sequences, the *Pmvs* or *Mpmvs* [112]. The *Pmv *and *Mpmv *proviruses that give rise to these recombinants have not been shown to be capable of producing infectious virus directly [113], but transmission of these ERVs and recombinant infectious P-MLVs can be accomplished through several mechanisms that are independent of XPR1. First, P-MLVs are generally transmitted in viremic mice as pseudotypes of E-MLVs and thus use mCAT-1 for entry [114, 115]. Also, homodimers representing the transcribed products of *Mpmv* and *Pmv *proviruses can be packaged into E-MLV virions, and these “mobilized” proviruses can infect cells, replicate in those new cells, and spread to other cells as pseudotyped virus [116]. Another transmission mechanism allows infectious, recombinant P-MLVs to use alternative receptors in the presence of the soluble RBD glycoprotein for that receptor. P-MLVs and entry defective E-MLVs, but not X-MLVs, can be “transactivated” in this way by E-MLV RBD [38, 108]. This transactivation process is controlled, at least in part, by the conserved Env residue H8 and has also been described for other viruses such as feline leukemia viruses [117]. While these mechanisms provide alternative routes for P-MLV transmission that are independent of the receptor, it should be noted that interference with the XPR1 receptor protects mice from P-MLV-induced disease [118] highlighting the crucial role of XPR1-mediated entry in the disease process.

## 11. Coevolution of XPR1 and XP-MLVs in *Mus*

The species distribution of the *Mus* XPR1 variants indicates that polymorphic, virus-restrictive receptors appeared when mice were exposed to XP-MLVs, especially X-MLVs (Figure 6). For most of the 8 million years of *Mus* evolution, species carried the permissive *Xpr1^sxv^* allele. Mice were subjected to XP-MLV infection about 0.5 MYA, and this exposure is marked by the acquisition of MLV ERVs in the 4 house mouse species [76, 119, 120]. *M. domesticus* carries P-MLV ERVs, whereas *M. castaneus*, *M. musculus,* and *M. molossinus* carry predominantly X-MLVs [76]. The common laboratory mouse strains, which are mosaics of these wild mouse species, generally carry multiple copies of X-MLVs and P-MLVs [121, 122]. The acquisition of these germline ERVs, and specifically X-MLVs, roughly coincides with the appearance of restrictive *Xpr1* alleles: *Xpr1^c^* in *M. castaneus*, *Xpr1^m^* in *M. molossinus* and *M. musculus*, and *Xpr1^n^* in laboratory mice (Figure 6). Each of these restrictive receptors carries a unique deletion in the XPR1 ECL4 (Figure 5) [17]. *M. domesticus*, the species carrying only inactive P-MLV ERVs, maintains the full-length, permissive *Xpr1^sxv^* receptor common to ancestral species of *Mus*. The fact that restrictive receptors have evolved in X-MLV infected mice suggests that this host pathogen interface has been an important evolutionary battleground. This also suggests that X-MLV infection is deleterious for mice, although the consequences of X-MLV infection have not yet been described because mouse gammaretroviruses have been studied largely in X-MLV-resistant laboratory mice. The discovery of mouse species and strains with XPR1 variants that efficiently support X-MLV entry now provides the basis for studies on pathogenesis of these viruses, and such studies have now been initiated [123].

## 12. CAT-1 and XPR1 Receptor Downregulation by Env Glycoproteins in *Mus*

Virus entry can be inhibited by receptor mutants but can also be blocked by members of a second set of genes found in E-MLV- or X-MLV-infected wild mice. This family of resistance genes governs production of MLV Env glycoproteins that are thought to restrict virus through receptor interference. These genes include *Fv4*, which blocks E-MLVs [124], and the genes *Rmcf* and *Rmcf2 *which restrict XP-MLVs and, in the case of *Rmcf*, inhibit P-MLV-induced disease [118, 124–126]. There is also evidence suggesting that additional *Rmcf*-like XPR1 receptor blocking genes are present in *M. castaneus *[127]. Specific ERVs have been mapped to 3 of these resistance genes, all of which are defective for virus production but have intact *env* genes. *Fv4* is a truncated provirus, *Rmcf *has a major deletion spanning *gag-pol* [124, 128], and *Rmcf2* has a stop codon that prematurely terminates integrase [125]. It has been proposed that the products of *Fv4*, *Rcmf*, and *Rcmf2* reduce or downregulate activity of their cognate receptors, and *Fv4* also has a defect in the fusion peptide of TM*env*, so incorporation of this Env into virions in virus-infected cells results in their reduced infectivity [129]. 

Interference genes that target both host range types are found in the Asian species *M. castaneus,* mice that are infected with X-MLVs as well as with E-MLVs that are related to the leukemogenic and neuropathic CasBrE E-MLV [76, 80, 130]. These mice rely on several survival strategies to mitigate the consequences of infection. In addition to their restrictive *Xpr1^c^* receptor, these mice carry *Fv4* as well as *Rmcf*-type XP-MLV interference genes. These interference genes likely arose in this species [80, 125]; CasBrE and *Fv4* have related Env genes (Figure 3) [130]. Transmission into other gene pools can proceed quickly for invasive genes like retrotransposons and for virus restriction genes that provide an immediate survival advantage. While the number and geographic distribution of *Rmcf*-type genes in Asian mice is not known, *Fv4* is found in AKV MLV-infected mice trapped in Japan (*M. molossinus)* and in Korea [80]. CasBrE and *Fv4* are both found in California mice, where Asian mice were likely introduced by the shipping trade [76, 82, 131]. The discovery of multiple interfering loci in infected mouse species and their geographic spread suggests that these coopted Env genes represent an effective survival mechanism. The general importance of this form of innate immunity is also illustrated by the fact that Env genes with similar antiviral functions have also been identified in chickens, sheep, and cats [132–134]. 

## 13. XPR1 Receptor Polymorphism and Entry Phenotypes in Non-*Mus* Species

**Figure 9:**
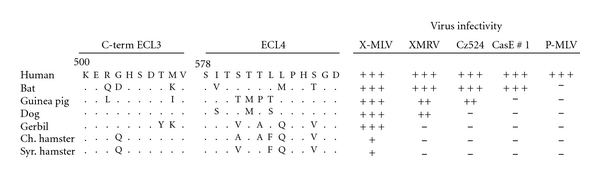
Susceptibility of various mammalian cells to XP-MLVs. Infectivity is measured as the log_10_ titer of FFU/100 *μ*L of viral Env pseudotypes carrying the LacZ reporter. Log_10_ titer: +++, >3; ++, 2-3; +, 1-2. Infectivity of Chinese hamster cells can be increased by treatment with glycosylation inhibitors. Amino acid sequences are shown for the receptor determining regions of ECL3 and ECL4.

The XP-MLVs are capable of infecting cells of other species, including humans (Figure 9). Cells of nearly all mammals are permissive to infection by X-MLVs, whereas a subset of these species is also susceptible to P-MLVs. This suggests that X-MLVs have less stringent receptor requirements than P-MLVs [17, 87, 88]. Some mammalian species show distinctive patterns of virus susceptibility not found in mice, for example, the restriction of P-MLVs and both wild mouse XP-MLVs by dog and buffalo cells (Figure 9) [17]. Analysis of mammalian XPR1 genes reveals significant sequence variability especially in the receptor determining ECL4, although this 13 residue segment contains 3 nonvariant residues, S578, T580, and G589. These conserved residues do not contribute to the receptor attachment site [17]. Further analysis of these functionally distinctive XPR1 genes may provide insight into the factors that facilitate transspecies transmission.

## 14. XMRV

Mice are important vectors of diseases that infect humans and their livestock [135], and MLV-infected house mouse species have a worldwide geographic distribution [136]. The horizontal transfer of infectious MLVs between individuals has been documented in wild mouse populations and in laboratory mice [82, 137], and MLV-related viral sequences, proteins, and antibodies have been reported in human blood donors and patients with prostate cancer and chronic fatigue syndrome [138–140]. An infectious virus first identified in prostate cancer patients, termed XMRV (xenotropic murine leukemia virus-related virus), shows close sequence homology with XP-MLVs [141], uses the XPR1 receptor [138], and has xenotropic host range [94]. Although XMRV origin by transspecies transmission is consistent with the evidence of MLV transmission between mice and evidence of transmission of mouse C-type viruses to other species [142–144], several recent studies on XMRV have implicated laboratory contamination [145–148]. Additional studies aiming to resolve the origins issue are focused on patient samples and the characterization of mice for XMRV-related sequences [149]. 

It is clear that XMRV differs from MLVs isolated from mice in several biological properties, including host range and receptor usage. The two critical residues for X-MLV entry in *Mus* XPR1, K500 and T582 [99], independently produce equivalent receptor determinants for X-MLV but not for XMRV [17] or for the wild mouse isolate CasE#1 [16]. While T582 but not K500 is required for CasE#1, XMRV preferentially relies on K500 [16, 17]. XMRV also differs from XP-MLVs in its ability to infect cells of different mammalian species. Although X-MLVs are able to infect all mammals, XMRV is uniquely restricted by cells of 3 species: Chinese hamster, Syrian hamster, and gerbil (Figure 9). For gerbil, this difference is likely attributable to XPR1 receptor polymorphism, as expression of the gerbil XPR1 receptor in heterologous cells reproduced the gerbil susceptibility pattern [17]. The restriction of XMRV in Syrian hamster BHK cells may involve other host factors. Expression of human *Xpr1* in these cells resulted in susceptibility to X-MLV but not XMRV, and analysis of interspecies somatic cell hybrids suggested that BHK cells lack a secondary factor needed for XMRV infection [150]. These results suggest that XMRV differs from other X-MLVs in its interaction with XPR1 receptor determinants and also suggest that XMRV may be uniquely dependent on an as yet unidentified receptor cofactor. Further studies with this virus may provide additional insight into xenotropism and the interactions and identity of viral and host proteins that direct entry.

## 15. Conclusions

Retrovirus entry is dependent on the presence and accessibility of specific cell surface receptors. Mutational changes in these receptors and in the receptor attachment sites in the virus Env can alter the very first step in the virus life cycle and can thus have profound consequences for virus replication. Inhibition of virus entry has been a particularly effective antiviral tactic in mice infected with MLVs as well as with other gammaretroviruses [151]. Entry is also the target of host restrictions in other species subject to retrovirus infection as shown by the discovery of interfering ERV Envs in multiple species [132–134] and by the discovery of inhibitory mutations in other receptors, such as the HIV-1 CCR5 coreceptor [152]. 

For the laboratory mouse MLVs, alterations in host receptors and/or virus Env can result in virus restriction, can alter the type and tempo of infection-induced pathology, and may also influence postentry events [153]. The interacting sites of these receptors and Env are highly polymorphic, as expected for coevolving entities in an “arms race” driven by sequential reciprocal adaptations. For the XP-MLVs and XMRV, the battleground at the cell surface has produced 5 functionally distinct receptors in mice and more than a half dozen distinctive host range virus variants, variants that interact with different but overlapping sets of determinants on the XPR1 receptor or rely on alternative mechanisms of transmission independent of XPR1. For the E-MLVs, CAT-1 shows more limited variation although multiple viral Env subtypes have evolved. The interacting interfaces of virus and host proteins are targeted not just by mutational changes but also by epigenetic modifications resulting from glycosylation and by a host defensive strategy that relies on co-option of germline *env *genes to interfere with virus infection. There is also evidence of additional host factors or co-factors that influence entry of the mouse gammaretroviruses, some of which are affected by glycosylation [150, 154]. Future studies on the mouse gammaretroviruses should identify other host factors involved in entry and trans-species transmission, should describe the consequences of X-MLV infection in mice permissive to the “xenotropic” viruses, and should further illuminate the coevolutionary paths of these pathogens and their hosts.

## Figures and Tables

**Table 1:** Functional variants of the XPR1 receptor in *Mus. *

	Infectivity^a^				Distribution^b^	
	X-MLV	P-MLV	CasE#1	Cz524	Common laboratory strains	*Mus *Species
*Xpr1^n^*	−	+++	−	−	Most	None
*Xpr1^sxv^*	+++	+++	+++	+++	F/St, LT, LP, SWR, SJL, SIM.R, SOD1	Most
*Xpr1^p^*	+++	−	+++	−	None	*M. pahari*
*Xpr1^c^*	++	−	−	+	None	*M. castaneus*
*Xpr1^m^*	++	−	−	−	None	*M. musculus,* *M. molossinus*

^a^Measured as log_10_ titer of FFU/100 *μ*L of viral Env pseudotypes carrying the LacZ reporter. Log_10_ titer: +++, >3; ++, 2-3; +, 1-2; −, 0-1.

^b^Determined for ~50 of the common strains of laboratory mice and *∼*20 of the 40 species of *Mus* [17, 95].

## References

[1] Stocking C, Kozak CA (2008). Murine endogenous retroviruses. *Cellular and Molecular Life Sciences*.

[2] Levy JA, Pincus T (1970). Demonstration of biological activity of a murine leukemia virus of New Zealand black mice. *Science*.

[3] Fischinger PJ, Nomura S, Bolognesi DP (1975). A novel murine oncornavirus with dual eco- and xenotropic properties. *Proceedings of the National Academy of Sciences of the United States of America*.

[4] Hartley JW, Wolford NK, Old LJ, Rowe WP (1977). A new class of murine leukemia virus associated with development of spontaneous lymphomas. *Proceedings of the National Academy of Sciences of the United States of America*.

[5] Kozak CA (1985). Susceptibility of wild mouse cells to exogenous infection with xenotropic leukemia viruses: control by a single dominant locus on chromosome 1. *Journal of Virology*.

[6] Tailor CS, Nouri A, Lee CG, Kozak C, Kabat D (1999). Cloning and characterization of a cell surface receptor for xenotropic and polytropic marine leukemia viruses. *Proceedings of the National Academy of Sciences of the United States of America*.

[7] Yang Y-L, Guo L, Xu S (1999). Receptors for polytropic and xenotropic mouse leukaemia viruses encoded by a single gene at *Rmc1*. *Nature Genetics*.

[8] Battini J-L, Rasko JEJ, Miller AD (1999). A human cell-surface receptor for xenotropic and polytropic murine leukemia viruses: possible role in G protein-coupled signal transduction. *Proceedings of the National Academy of Sciences of the United States of America*.

[9] Battini JL, Danos O, Heard JM (1995). Receptor-binding domain of murine leukemia virus envelope glycoproteins. *Journal of Virology*.

[10] Heard JM, Danos O (1991). An amino-terminal fragment of the Friend murine leukemia virus envelope glycoprotein binds the ecotropic receptor. *Journal of Virology*.

[11] Battini JL, Heard JM, Danos O (1992). Receptor choice determinants in the envelope glycoproteins of amphotropic, xenotropic, and polytropic murine leukemia viruses. *Journal of Virology*.

[12] Levy JA (1978). Xenotropic type C viruses. *Current Topics in Microbiology and Immunology*.

[13] Eiden MV, Farrell K, Warsowe J, Mahan LC, Wilson CA (1993). Characterization of a naturally occurring ecotropic receptor that does not facilitate entry of all ecotropic murine retroviruses. *Journal of Virology*.

[14] Kozak CA (1983). Genetic mapping of a mouse chromosomal locus required for mink cell focus-forming virus replication. *Journal of Virology*.

[15] Lyu MS, Kozak CA (1996). Genetic basis for resistance to polytropic murine leukemia viruses in the wild mouse species *Mus castaneus*. *Journal of Virology*.

[16] Yan Y, Knoper RC, Kozak CA (2007). Wild mouse variants of envelope genes of xenotropic/polytropic mouse gammaretroviruses and their XPR1 receptors elucidate receptor determinants of virus entry. *Journal of Virology*.

[17] Yan Y, Liu Q, Wollenberg K, Martin C, Buckler-White A, Kozak CA (2010). Evolution of functional and sequence variants of the mammalian XPR1 receptor for mouse xenotropic gammaretroviruses and the human-derived retrovirus XMRV. *Journal of Virology*.

[18] Albritton LM, Tseng L, Scadden D, Cunningham JM (1989). A putative murine ecotropic retrovirus receptor gene encodes a multiple membrane-spanning protein and confers susceptibility to virus infection. *Cell*.

[19] Kim JW, Closs EI, Albritton LM, Cunningham JM (1991). Transport of cationic amino acids by the mouse ecotropic retrovirus receptor. *Nature*.

[20] Wang H, Kavanaugh MP, North RA, Kabat D (1991). Cell-surface receptor for ecotropic murine retroviruses is a basic amino-acid transporter. *Nature*.

[21] Overbaugh J, Miller AD, Eiden MV (2001). Receptors and entry cofactors for retroviruses include single and multiple transmembrane-spanning proteins as well as newly described glycophosphatidylinositol-anchored and secreted proteins. *Microbiology and Molecular Biology Reviews*.

[22] Tailor CS, Lavillette D, Marin M, Kabat D (2003). Cell surface receptors for gammaretroviruses. *Current Topics in Microbiology and Immunology*.

[23] Ericsson TA, Takeuchi Y, Templin C (2003). Identification of receptors for pig endogenous retrovirus. *Proceedings of the National Academy of Sciences of the United States of America*.

[24] Hein S, Prassolov V, Zhang Y (2003). Sodium-dependent myo-inositol transporter 1 is a cellular receptor for Mus cervicolor M813 murine leukemia virus. *Journal of Virology*.

[25] Mendoza R, Anderson MM, Overbaugh J (2006). A putative thiamine transport protein is a receptor for feline leukemia virus subgroup A. *Journal of Virology*.

[26] Miller AD, Bergholz U, Ziegler M, Stocking C (2008). Identification of the myelin protein plasmolipin as the cell entry receptor for Mus caroli endogenous retrovirus. *Journal of Virology*.

[27] Albritton LM, Kim JW, Tseng L, Cunningham JM (1993). Envelope-binding domain in the cationic amino acid transporter determines the host range of ecotropic murine retroviruses. *Journal of Virology*.

[28] Yoshimoto T, Yoshimoto E, Meruelo D (1993). Identification of amino acid residues critical for infection with ecotropic murine leukemia retrovirus. *Journal of Virology*.

[29] Kim JW, Cunningham JM (1993). N-linked glycosylation of the receptor for murine ecotropic retroviruses is altered in virus-infected cells. *Journal of Biological Chemistry*.

[30] Pizzato M, Marlow SA, Blair ED, Takeuchi Y (1999). Initial binding of murine leukemia virus particles to cells does not require specific Env-receptor interaction. *Journal of Virology*.

[31] Jinno-Oue A, Oue M, Ruscetti SK (2001). A unique heparin-binding domain in the envelope protein of the neuropathogenic PVC-211 murine leukemia virus may contribute to its brain capillary endothelial cell tropism. *Journal of Virology*.

[32] Kumar P, Nachagari D, Fields C, Franks J, Albritton LM (2007). Host cell cathepsins potentiate moloney murine leukemia virus infection. *Journal of Virology*.

[33] Park BH, Matuschke B, Lavi E, Gaulton GN (1994). A point mutation in the *env* gene of a murine leukemia virus induces syncytium formation and neurologic disease. *Journal of Virology*.

[34] Mackrell AJ, Soong NW, Curtis CM, French Anderson W (1996). Identification of a subdomain in the Moloney murine leukemia virus envelope protein involved in receptor binding. *Journal of Virology*.

[35] Davey RA, Zuo Y, Cunningham JM (1999). Identification of a receptor-binding pocket on the envelope protein of Friend murine leukemia virus. *Journal of Virology*.

[36] Fass D, Davey RA, Hamson CA, Kim PS, Cunningham JM, Berger JM (1997). Structure of a murine leukemia virus receptor-binding glycoprotein 2.0 angstrom resolution. *Science*.

[37] Bae Y, Kingsman SM, Kingsman AJ (1997). Functional dissection of the Moloney murine leukemia virus envelope protein gp70. *Journal of Virology*.

[38] Lavillette D, Ruggieri A, Russell SJ, Cosset FL (2000). Activation of a cell entry pathway common to type C mammalian retroviruses by soluble envelope fragments. *Journal of Virology*.

[39] Zavorotinskaya T, Albritton LM (1999). Suppression of a fusion defect by second site mutations in the ecotropic murine leukemia virus surface protein. *Journal of Virology*.

[40] Lavillette D, Maurice M, Roche C, Russell SJ, Sitbon M, Cosset FL (1998). A proline-rich motif downstream of the receptor binding domain modulates conformation and fusogenicity of murine retroviral envelopes. *Journal of Virology*.

[41] Pinter A, Kopelman R, Li Z, Kayman SC, Sanders DA (1997). Localization of the labile disulfide bond between SU and TM of the murine leukemia virus envelope protein complex to a highly conserved CWLC motif in SU that resembles the active-site sequence of thiol-disulfide exchange enzymes. *Journal of Virology*.

[42] Lavillette D, Boson B, Russell SJ, Cosset FL (2001). Activation of membrane fusion by murine leukemia viruses is controlled in *cis* or in *trans* by interactions between the receptor-binding domain and a conserved disulfide loop of the carboxy terminus of the surface glycoprotein. *Journal of Virology*.

[43] Burkhart MD, D'Agostino P, Kayman SC, Pinter A (2005). Involvement of the C-terminal disulfide-bonded loop of murine leukemia virus SU protein in a postbinding step critical for viral entry. *Journal of Virology*.

[44] Yan Y, Kozak CA (2008). Novel postentry resistance to AKV ecotropic mouse gammaretroviruses in the African pygmy mouse, *Mus minutoides*. *Journal of Virology*.

[45] Jung YT, Kozak CA (2003). Generation of novel syncytium-inducing and host range variants of ecotropic Moloney murine leukemia virus in *Mus spicilegus*. *Journal of Virology*.

[46] Ishimoto A (1985). Infectivity of friend murine leukemia virus for hamster cells. *Journal of the National Cancer Institute*.

[47] Masuda M, Hanson CA, Hoffman PM (1996). Analysis of the unique hamster cell tropism of ecotropic murine leukemia virus PVC-211. *Journal of Virology*.

[48] Jung YT, Wu T, Kozak CA (2004). Novel host range and cytopathic variant of ecotropic Friend murine leukemia virus. *Journal of Virology*.

[49] Yan Y, Jung YT, Wu T, Kozak CA (2008). Role of receptor polymorphism and glycosylation in syncytium induction and host range variation of ecotropic mouse gammaretroviruses. *Retrovirology*.

[50] Weller SK, Temin HM (1981). Cell killing by avian leukosis viruses. *Journal of Virology*.

[51] Rohn JL, Moser MS, Gwynn SR, Baldwin DN, Overbaugh J (1998). In vivo evolution of a novel, syncytium-inducing and cytopathic feline leukemia virus variant. *Journal of Virology*.

[52] Cheng-Mayer C, Seto D, Tateno M, Levy JA (1988). Biologic features of HIV-1 that correlate with virulence in the host. *Science*.

[53] Temin HM (1988). Mechanisms of cell killing/cytopathic effects by nonhuman retroviruses. *Reviews of Infectious Diseases*.

[54] Murphy SL, Chung-Landers M, Honczarenko M, Gaulton GN (2006). Linkage of reduced receptor affinity and superinfection to pathogenesis of TR1.3 murine leukemia virus. *Journal of Virology*.

[55] Poliquin L, Bergeron D, Fortier JL, Paquette Y, Bergeron R, Rassart E (1992). Determinants of thymotropism in Kaplan radiation leukemia virus and nucleotide sequence of its envelope region. *Journal of Virology*.

[56] Murphy SL, Honczarenko MJ, Dugger NV, Hoffman PM, Gaulton GN (2004). Disparate regions of envelope protein regulate syncytium formation versus spongiform encephalopathy in neurological disease induced by murine leukemia virus TR. *Journal of Virology*.

[57] DesGroseillers L, Barrette M, Jolicoeur P (1984). Physical mapping of the paralysis-inducing determinant of a wild mouse ecotropic neurotropic retrovirus. *Journal of Virology*.

[58] Li Y, Cardona SM, Traister RS, Lynch WP (2011). Retrovirus-induced spongiform neurodegeneration is mediated by unique central nervous system viral targeting and expression of Env alone. *Journal of Virology*.

[59] Masuda M, Hanson CA, Alvord WG, Hoffman PM, Ruscetti SK, Masuda M (1996). Effects of subtle changes in the SU protein of ecotropic murine leukemia virus on its brain capillary endothelial cell tropism and interference properties. *Virology*.

[60] Olofsson S, Bergström T (2005). Glycoconjugate glycans as viral receptors. *Annals of Medicine*.

[61] Wang H, Klamo E, Kuhmann SE, Kozak SL, Kavanaugh MP, Kabat D (1996). Modulation of ecotropic murine retroviruses by N-linked glycosylation of the cell surface receptor/amino acid transporter. *Journal of Virology*.

[62] Eiden MV, Farrell K, Wilson CA (1994). Glycosylation-dependent inactivation of the ecotropic murine leukemia virus receptor. *Journal of Virology*.

[63] Wilson CA, Eiden MV (1991). Viral and cellular factors governing hamster cell infection by murine and gibbon ape leukemia viruses. *Journal of Virology*.

[64] Miller DG, Miller AD (1992). Tunicamycin treatment of CHO cells abrogates multiple blocks to retrovirus infection, one of which is due to a secreted inhibitor. *Journal of Virology*.

[65] Tavoloni N, Rudenholz A (1997). Variable transduction efficiency of murine leukemia retroviral vector on mammalian cells: role of cellular glycosylation. *Virology*.

[66] Kubo Y, Ono T, Ogura M, Ishimoto A, Amanuma H (2002). A glycosylation-defective variant of the ecotropic murine retrovirus receptor is expressed in rat XC cells. *Virology*.

[67] Kubo Y, Ishimoto A, Amanuma H (2003). N-linked glycosylation is required for XC cell-specific syncytium formation by the peptide-containing envelope protein of ecotropic murine leukemia viruses. *Journal of Virology*.

[68] Ogert RA, Lee MK, Ross W, Buckler-White A, Martin MA, Cho MW (2001). N-linked glycosylation sites adjacent to and within the V1/V2 and the V3 loops of dualtropic human immunodeficiency virus type 1 isolate DH12 gp120 affect coreceptor usage and cellular tropism. *Journal of Virology*.

[69] Schultz AM, Oroszlan S (1979). Tunicamycin inhibits glycosylation of precursor polyprotein encoded by env gene of Rauscher murine leukemia virus. *Biochemical and Biophysical Research Communications*.

[70] Felkner RH, Roth MJ (1992). Mutational analysis of the N-linked glycosylation sites of the SU envelope protein of Moloney murine leukemia virus. *Journal of Virology*.

[71] Kayman SC, Kopelman R, Projan S, Kinney DM, Pinter A (1991). Mutational analysis of N-linked glycosylation sites of Friend murine leukemia virus envelope protein. *Journal of Virology*.

[72] Li Z, Pinter A, Kayman SC (1997). The critical N-linked glycan of murine leukemia virus envelope protein promotes both folding of the C-terminal domains of the precursor polyprotein and stability of the postcleavage envelope complex. *Journal of Virology*.

[73] Andersen KB (1994). A domain of murine retrovirus surface protein gp70 mediates cell fusion, as shown in a novel SC-1 cell fusion system. *Journal of Virology*.

[74] Battini JL, Kayman SC, Pinter A, Heard JM, Danos O (1994). Role of N-linked glycosylation in the activity of the Friend murine leukemia virus SU protein receptor-binding domain. *Virology*.

[75] Knoper RC, Ferrarone J, Yan Y, Lafont BAP, Kozak CA (2009). Removal of either N-glycan site from the envelope receptor binding domain of Moloney and Friend but not AKV mouse ecotropic gammaretroviruses alters receptor usage. *Virology*.

[76] Kozak CA, O'Neill RR (1987). Diverse wild mouse origins of xenotropic, mink cell focus-forming, and two types of ecotropic proviral genes. *Journal of Virology*.

[77] Jenkins NA, Copeland NG, Taylor BA, Lee BK (1982). Organization, distribution, and stability of endogenous ecotropic murine leukemia virus DNA sequences in chromosomes of *Mus musculus*. *Journal of Virology*.

[78] Kozak CA, Rowe WP (1982). Genetic mapping of ecotropic murine leukemia virus-inducing loci in six inbred strains. *Journal of Experimental Medicine*.

[79] Kozak CA, Ruscetti S, Levy J (1994). Retroviruses in rodents. *The Retroviridae*.

[80] Inaguma Y, Miyashita N, Moriwaki K (1991). Acquisition of two endogenous ecotropic murine leukemia viruses in distinct Asian wild mouse populations. *Journal of Virology*.

[81] Gardner MB, Klement V, Rongey RR (1976). Type C virus expression in lymphoma paralysis prone wild mice. *Journal of the National Cancer Institute*.

[82] Gardner MB, Chiri A, Dougherty MF (1979). Congenital transmission of murine leukemia virus from wild mice prone to the development of lymphoma and paralysis. *Journal of the National Cancer Institute*.

[83] Rassart E, Nelbach L, Jolicoeur P (1986). Cas-Br-E murine leukemia virus: sequencing of the paralytogenic regions of its genome and derivation of specific probes to study its origin and the structure of its recombinant genomes in leukemic tissues. *Journal of Virology*.

[84] Orth A, Adama T, Din W, Bonhomme F, Singh RS (1998). Natural hybridation between two domestic mouse subspecies, *Mus musculus domesticus* and *Mus musculus castaneus* near by Lake Casitas, California. *Genome*.

[85] Voytek P, Kozak CA (1989). Nucleotide sequence and a mode of transmission of the wild mouse ecotropic virus, HoMuLV. *Virology*.

[86] Lander MR, Chattopadhyay SK (1984). A *mus dunni* cell line that lacks sequences closely related to endogenous murine leukemia viruses and can be infected by ecotropic, amphotropic, xenotropic, and mink cell focus-forming viruses. *Journal of Virology*.

[87] Levy JA (1975). Host range of murine xenotropic virus: replication in avian cells. *Nature*.

[88] Oie HK, Russell EK, Dotson JH (1976). Host range properties of murine xenotropic and ecotropic type C viruses. *Journal of the National Cancer Institute*.

[89] Cloyd MW, Thompson MM, Hartley JW (1985). Host range of mink cell focus-inducing viruses. *Virology*.

[90] Vogt M, Haggblom C, Swift S, Haas M (1985). Envelope gene and long terminal repeat determine the different biological properties of Rauscher, Friend, and Moloney mink cell focus-inducing viruses. *Journal of Virology*.

[91] Chatis PA, Holland CA, Silver JE (1984). A 3' end fragment encompassing the transcriptional enhancers of nondefective Friend virus confers erythroleukemogenicity on Moloney leukemia virus. *Journal of Virology*.

[92] Ishimoto A, Adachi A, Sakai K, Matsuyama M (1985). Long terminal repeat of friend-MCF virus contains the sequence responsible for erythroid leukemia. *Virology*.

[93] Bahrami S, Duch M, Pedersen FS (2004). Change of tropism of SL3-2 murine leukemia virus, using random mutational libraries. *Journal of Virology*.

[94] Yan Y, Liu Q, Kozak CA (2009). Six host range variants of the xenotropic/polytropic gammaretroviruses define determinants for entry in the XPR1 cell surface receptor. *Retrovirology*.

[95] Baliji S, Liu Q, Kozak CA (2010). Common inbred strains of the laboratory mouse that are susceptible to infection by mouse xenotropic gammaretroviruses and the human-derived retrovirus XMRV. *Journal of Virology*.

[96] Hartley JW, Rowe WP (1975). Clonal cell lines from a feral mouse embryo which lack host range restrictions for murine leukemia viruses. *Virology*.

[97] Chesebro B, Wehrly K (1985). Different murine cells lines manifest unique patterns of interference to superinfection by murine leukemia viruses. *Virology*.

[98] Sharma P, Patntirapong S, Hann S, Hauschka PV (2010). RANKL-RANK signaling regulates expression of xenotropic and polytropic virus receptor (XPR1) in osteoclasts. *Biochemical and Biophysical Research Communications*.

[99] Marin M, Tailor CS, Nouri A, Kozak SL, Kabat D (1999). Polymorphisms of the cell surface receptor control mouse susceptibilities to xenotropic and polytropic leukemia viruses. *Journal of Virology*.

[100] Morse HCI, Morse HC (1978). Introduction. *Origins of Inbred Mice*.

[101] Yang H, Bell TA, Churchill GA, Pardo-Manuel De Villena F (2007). On the subspecific origin of the laboratory mouse. *Nature Genetics*.

[102] Van Hoeven NS, Miller AD (2005). Use of different but overlapping determinants in a retrovirus receptor accounts for non-reciprocal interference between xenotropic and polytropic murine leukemia viruses. *Retrovirology*.

[103] Brown JK, Fung C, Tailor CS (2006). Comprehensive mapping of receptor-functioning domains in feline leukemia virus subgroup C receptor FLVCR1. *Journal of Virology*.

[104] Farrell KB, Russ JL, Murthy RK, Eiden MV (2002). Reassessing the role of region A in Pit1-mediated viral entry. *Journal of Virology*.

[105] Manel N, Battini JL, Sitbon M (2005). Human T cell leukemia virus envelope binding and virus entry are mediated by distinct domains of the glucose transporter GLUT1. *Journal of Biological Chemistry*.

[106] Barnett AL, Davey RA, Cunningham JM (2001). Modular organization of the Friend murine leukemia virus envelope protein underlies the mechanism of infection. *Proceedings of the National Academy of Sciences of the United States of America*.

[107] Barnett AL, Cunningham JM (2001). Receptor binding transforms the surface subunit of the mammalian C-type retrovirus envelope protein from an inhibitor to an activator of fusion. *Journal of Virology*.

[108] Wensel DL, Li W, Cunningham JM (2003). A virus-virus interaction circumvents the virus receptor requirement for infection by pathogenic retroviruses. *Journal of Virology*.

[109] Herr W, Gilbert W (1984). Free and integrated recombinant murine leukemia virus DNAs appear in preleukemic thymuses of AKR/J mice. *Journal of Virology*.

[110] Yoshimura FK, Wang T, Yu F, Kim HRC, Turner JR (2000). Mink cell focus-forming murine leukemia virus infection induces apoptosis of thymic lymphocytes. *Journal of Virology*.

[111] Nanua S, Yoshimura FK (2004). Mink epithelial cell killing by pathogenic murine leukemia viruses involves endoplasmic reticulum stress. *Journal of Virology*.

[112] Stoye JP, Moroni C, Coffin JM (1991). Virological events leading to spontaneous AKR thymomas. *Journal of Virology*.

[113] Jern P, Stoye JP, Coffin JM (2007). Role of APOBEC3 in genetic diversity among endogenous murine leukemia viruses. *PLoS Genetics*.

[114] Fischinger PJ, Blevins CS, Dunlop NM (1978). Genomic masking of nondefective recombinant murine leukemia virus in Moloney virus stocks. *Science*.

[115] Lavignon M, Evans L (1996). A multistep process of leukemogenesis in moloney murine leukemia virus-infected mice that is modulated by retroviral pseudotyping and interference. *Journal of Virology*.

[116] Evans LH, Alamgir AS, Owens N (2009). Mobilization of endogenous retroviruses in mice after infection with an exogenous retrovirus. *Journal of Virology*.

[117] Anderson MM, Lauring AS, Burns CC (2000). Identification of a cellular cofactor required for infection by feline leukemia virus. *Science*.

[118] Ruscetti S, Davis L, Feild J, Oliff A (1981). Friend murine leukemia virus-induced leukemia is associated with the formation of mink cell focus-inducing viruses and is blocked in mice expressing endogenous mink cell focus-inducing xenotropic viral envelope genes. *Journal of Experimental Medicine*.

[119] Boursot P, Din W, Anand R (1996). Origin and radiation of the house mouse: mitochondrial DNA phylogeny. *Journal of Evolutionary Biology*.

[120] Din W, Anand R, Boursot P (1996). Origin and radiation of the house mouse: clues from nuclear genes. *Journal of Evolutionary Biology*.

[121] O'Neill RR, Khan AS, Hoggan MD (1986). Specific hybridization probes demonstrate fewer xenotropic than mink cell focus-forming murine leukemia virus *env*-related sequences in DNAs from inbred laboratory mice. *Journal of Virology*.

[122] Frankel WN, Stoye JP, Taylor BA, Coffin JM (1990). A linkage map of endogenous murine leukemia proviruses. *Genetics*.

[123] Sakuma T, Tonne JM, Squillace KA (2011). Early events in retrovirus XMRV infection of the wild-derived mouse *Mus pahari*. *Journal of Virology*.

[124] Ikeda H, Laigret F, Martin MA, Repaske R (1985). Characterization of a molecularly cloned retroviral sequence associated with *Fv-4* resistance. *Journal of Virology*.

[125] Wu T, Yan Y, Kozak CA (2005). *Rmcf2*, a xenotropic provirus in the Asian mouse species *Mus castaneus*, blocks infection by polytropic mouse gammaretroviruses. *Journal of Virology*.

[126] Bassin RH, Ruscetti S, Ali I, Haapala DK, Rein A (1982). Normal DBA/2 mouse cells synthesize a glycoprotein which interferes with MCF virus infection. *Virology*.

[127] Kozak CA (2010). The mouse "xenotropic" gammaretroviruses and their XPR1 receptor. *Retrovirology*.

[128] Jung YT, Lyu MS, Buckler-White A, Kozak CA (2002). Characterization of a polytropic murine leukemia virus proviral sequence associated with the virus resistance gene *Rmcf* of *DBA/2 mice*. *Journal of Virology*.

[129] Taylor GM, Gao Y, Sanders DA (2001). Fv-4: identification of the defect in Env and the mechanism of resistance to ecotropic murine leukemia virus. *Journal of Virology*.

[130] Ikeda H, Kato K, Kitani H (2001). Virological properties and nucleotide sequences of cas-E-type endogenous ecotropic murine leukemia viruses in south asian wild mice, *Mus musculus castaneus*. *Journal of Virology*.

[131] Gardner MB, Rasheed S, Pal BK (1980). Akvr-1, a dominant murine leukemia virus restriction gene, is polymorphic in leukemia-prone wild mice. *Proceedings of the National Academy of Sciences of the United States of America*.

[132] Robinson HL, Astrin SM, Senior AM, Salazar FH (1981). Host susceptibility to endogenous viruses: defective, glycoprotein-expressing proviruses interfere with infections. *Journal of Virology*.

[133] Spencer TE, Mura M, Gray CA, Griebel PJ, Palmarini M (2003). Receptor usage and fetal expression of ovine endogenous betaretroviruses: implications for coevolution of endogenous and exogenous retroviruses. *Journal of Virology*.

[134] McDougall AS, Terry A, Tzavaras T, Cheney C, Rojko J, Neil JC (1994). Defective endogenous proviruses are expressed in feline lymphoid cells: Evidence for a role in natural resistance to subgroup B feline leukemia viruses. *Journal of Virology*.

[135] Weber WJ (1982). *Diseases Transmitted by Rats and Mice*.

[136] Marshall J, Foster SJHL, Fox JG (1981). Taxonomy. *The Mouse in Biomedical Research*.

[137] Portis JL, McAtee FJ, Hayes SF (1987). Horizontal transmission of murine retroviruses. *Journal of Virology*.

[138] Dong B, Kim S, Hong S (2007). An infectious retrovirus susceptible to an IFN antiviral pathway from human prostate tumors. *Proceedings of the National Academy of Sciences of the United States of America*.

[139] Lombardi VC, Ruscetti FW, das Gupta J (2009). Detection of an infectious retrovirus, XMRV, in blood cells of patients with chronic fatigue syndrome. *Science*.

[140] Knouf EC, Metzger MJ, Mitchell PS (2009). Multiple integrated copies and high-level production of the human retrovirus XMRV (Xenotropic Murine leukemia virus-Related Virus) from 22Rv1 prostate carcinoma cells. *Journal of Virology*.

[141] Urisman A, Molinaro RJ, Fischer N (2006). Identification of a novel gammaretrovirus in prostate tumors of patients homozygous for R462Q RNASEL variant. *PLoS Pathogens*.

[142] Benveniste RE, Todaro GJ (1973). Homology between type C viruses of various species as determined by molecular hybridization. *Proceedings of the National Academy of Sciences of the United States of America*.

[143] Benveniste RE, Heinemann R, Wilson GL (1974). Detection of baboon type C viral sequences in various primate tissues by molecular hybridization. *Journal of Virology*.

[144] Lieber MM, Sherr CJ, Todaro GJ (1975). Isolation from the Asian mouse *Mus caroli* of an endogenous type C virus related to infectious primate type C viruses. *Proceedings of the National Academy of Sciences of the United States of America*.

[145] Hue S, Gray ER, Gall A (2010). Disease-associated XMRV sequences are consistent with laboratory contamination. *Retrovirology*.

[146] Robinson MJ, Erlwein OW, Kaye S (2010). Mouse DNA contamination in human tissue tested for XMRV. *Retrovirology*.

[147] Oakes B, Tai AK, Cingoz O (2010). Contamination of human DNA samples with mouse DNA can lead to false detection of XMRV-like sequences. *Retrovirology*.

[148] Sato E, Furuta RA, Miyazawa T (2010). An endogenous murine leukemia viral genome contaminant in a commercial RT-PCR Kit is amplified using standard primers for XMRV. *Retrovirology*.

[149] Paprotka T, Delviks-Frankenberry KA, Cingöz O (2011). Recombinant origin of the retrovirus XMRV. *Science*.

[150] Xu WQ, Eiden MV (2011). Primate gammaretroviruses require an ancillary factor not required for murine gammaretroviruses to infect BHK cells. *Journal of Virology*.

[151] Wilson CA, Farrell KB, Eiden MV (1994). Comparison of cDNAs encoding the gibbon ape leukaemia virus receptor from susceptible and non-susceptible murine cells. *Journal of General Virology*.

[152] Liu R, Paxton WA, Choe S (1996). Homozygous defect in HIV-1 coreceptor accounts for resistance of some multiply-exposed individuals to HIV-1 infection. *Cell*.

[153] Oliveira NMM, Trikha R, McKnight Á (2010). A novel envelope mediated post entry restriction of murine leukaemia virus in human cells is Ref1/TRIM5*α* independent. *Retrovirology*.

[154] Miller DG, Miller AD (1993). Inhibitors of retrovirus infection are secreted by several hamster cell lines and are also present in hamster sera. *Journal of Virology*.

